# QT Prolongation due to Graves' Disease

**DOI:** 10.1155/2017/7612748

**Published:** 2017-01-05

**Authors:** Zain Kulairi, Nisha Deol, Renee Tolly, Rohan Manocha, Maliha Naseer

**Affiliations:** Department of Internal Medicine, Crittenton Hospital Medical Center, Wayne State University, Rochester, MI 48307, USA

## Abstract

Hyperthyroidism is a highly prevalent disease affecting over 4 million people in the US. The disease is associated with many cardiac complications including atrial fibrillation and also less commonly with ventricular tachycardia and fibrillation. Many cardiac pathologies have been extensively studied; however, the relationship between hyperthyroidism and rate of ventricular repolarization manifesting as a prolonged QTc interval is not well known. Prolonged QTc interval regardless of thyroid status is a risk factor for cardiovascular mortality and life-threatening ventricular arrhythmia. The mechanism regarding the prolongation of the QT interval in a hyperthyroid patient has not been extensively investigated although its clinical implications are relevant. Herein, we describe a case of prolonged QTc in a patient who presented with signs of hyperthyroidism that was corrected with return to euthyroid status.

## 1. Background

Hyperthyroidism is a high frequency endocrine disorder with a prevalence of around 1.3 percent in the general population and is known to be associated with cardiovascular complications. Individuals with hyperthyroidism have a 20% increase in mortality with the predominant etiology being cardiac related [1]. The most common cardiac complication is atrial fibrillation which occurs in approximately 10–25% of overtly hyperthyroid patients [1]. Not as common but still present is the frequency of ventricular tachycardia or ventricular fibrillation leading to sudden cardiac death. Cardiovascular hemodynamic changes which are commonly linked to such complications include increased contractility, increased preload, and decreased systemic vascular resistance, all resulting in increased cardiac output. In a small percentage of patients with hyperthyroidism, thyrotoxic cardiomyopathy can occur which has also been linked to high mortality [1].

Ventricular repolarization is quantified by the QT interval corrected by heart rate (QTc) [2]. The prolongation of the QT interval has been associated with tachyarrhythmia which may lead to ventricular fibrillation and sudden cardiac death [3]. A prospective population-based cohort study with 3,105 men and 4,878 women showed abnormal prolonged QTc interval was associated with a threefold increase risk of sudden cardiac death using Cox proportional hazards analysis [3]. Therefore the significance of observing QTc intervals within hyperthyroidism patients could prevent life-threatening consequences.

## 2. Case Report

A 29-year-old male with no significant past medical history presented with palpitations that had been continuous throughout the day and night for the past two weeks. He stated he felt as though his pulse was bounding. He complained of sleep disturbances, weight loss, and heat intolerance. Patient denied syncope or loss of consciousness. There is no family history of heart disease or sudden death in the family. Patient is not on any medication and he drinks only 1 cup of coffee per day and no other caffeinated beverages. On examination vital signs were within normal limits with a heart rate of 90 bpm and bounding pulse; cardiac examination showed a regular rate and rhythm with no murmurs, rubs, or gallops. Otherwise physical examination was unremarkable. Electrocardiogram revealed sinus rhythm and a prolonged QTc of 509. No previous EKG was available for comparison. CBC and BMP were all within normal limits with exception of a TSH < 0.01, T3 of 7.58 (normal lab value 2–4.4), and T4 of 2.71 (normal lab value 0.93–1.7). Repeat electrocardiogram revealed a QTc of 510. Ultrasound of thyroid was negative for nodularity. Radioactive iodine showed increased uptake in diffuse pattern. Echocardiogram was within normal limits showing no structural abnormalities. Holter monitor revealed no arrhythmia. The patient was diagnosed with Graves' disease in which propranolol and methimazole were started.

The patient was followed up in 3 months and a repeat level revealed TSH of 4.42, T3 of 3.72, and T4 of 1.25. All the symptoms of palpitations and heat intolerance have resolved. A repeat electrocardiogram was also done which revealed normal sinus rhythm with QTc of 410.

## 3. Discussion

Numerous studies including one by Straus et al. found that in a retrospective study of 65 patients the mean QTc interval in hyperthyroid patients was 434.16 msec and 411.31 msec in control subjects. Furthermore, there was a correlation between the free T4 and the degree of QT prolongation; patients with higher levels of free T4 had longer QTc duration. Our patient presented with signs of hyperthyroidism including palpitations which warranted an electrocardiogram. Based on the fact that previous EKGs in our patient demonstrated normal QTc intervals, this incidental finding on EKG was surprising. However, correction of patient's hyperthyroid state led to improvement of QTc and also decreased risk of potential ventricular arrhythmia and sudden cardiac death.

The correlation of hyperthyroidism and QTc prolongation is not well understood; however there are a few studies which have looked into the mechanism. One postulated theory is the effect of thyroid hormone on the cardiac myocyte Na/K1 ATPase. It is proposed that increased activity in this receptor secondary to the action of T4 corresponds with increased intracellular potassium causing hyperpolarization of the membrane and prolonging repolarization represented as a prolonged QTc. A study completed with rats, showed hearts that were transplanted and treated with T4 displayed increased gene expression of the NA/K ATPase independent of the increased hemodynamic status and cardiac workload that occurs in hyperthyroid states [4]. These findings provide further evidence of the direct dose dependent effect of T4 on intracellular potassium and thus ventricular repolarization manifesting as a prolonged QTc. It should also be noted that correction of free T4 was directly associated with correction of the QTc interval [3] and conversely that higher levels of free T4 were related to greater increases in QTc interval. The clinical relevance applies to the fact that patients that present with acute thyrotoxicosis have a higher likelihood of suffering from tachyarrhythmia. In our patient subsequent transition from hyperthyroid to euthyroid state was associated with change in QTc interval from 510 to 410.

Lastly, the heart has clinically and experimentally been proven to be a target organ for thyroid hormone. Data has shown 8,435 responsive genes to thyroid hormone which are involved in cardiac muscle activity [5]. Studies have also provided evidence that isoforms of ligand-modulated transcription factor T3 receptors (TR) *α*1 and *β*1 are present in the heart [5, 6]. These isoforms may provide further information regarding the possibility of genetic predisposition to cardiac conduction abnormalities associated with hyperthyroidism and may further explain why some hyperthyroid patients manifest prolonged QTc intervals and others do not. Furthermore, varying isoforms may shed light regarding why both hyperthyroid and hypothyroid states have been associated with QT abnormalities.

## 4. Conclusion

We report a case of prolonged QTc found incidentally in a patient with clinical signs and symptoms of hyperthyroidism that was diagnosed with Graves' disease. The QTc interval was corrected to a normal range as the patient became euthyroid. Electrocardiogram with special attention to the QTc interval should be a routine investigation on initial presentation and patient follow-up visits in patients that are in a hyperthyroid state until T4 is corrected. This may also shed light on etiology of sudden cardiac death in patients with known hyperthyroidism especially those of new onset without adequate correction of T4. This may also be especially pertinent for presentations of acute thyrotoxicosis in new onset hyperthyroid patients that present to the emergency department. Additionally, closer attention should be directed towards avoiding commonly utilized medications that may also exacerbate the QTc prolongation through an additive effect in patients transitioning from hyperthyroid to euthyroid state. Further investigation is required regarding the links between hyperthyroidism and QTc prolongation.

## Abbreviations

EKG: Electrocardiogram
T3: Triiodothyronine
T4: Thyroxine
TSH: Thyroid stimulating hormone
QTc: Corrected QT interval
CBC: Complete blood count
BMP: Basic metabolic panel.
